# COVID-19 pneumonia chest radiographic severity score: variability assessment among experienced and in-training radiologists and creation of a multireader composite score database for artificial intelligence algorithm development

**DOI:** 10.1259/bjr.20211028

**Published:** 2022-05-05

**Authors:** Marly van Assen, Mohammadreza Zandehshahvar, Hossein Maleki, Yashar Kiarashi, Timothy Arleo, Arthur E. Stillman, Peter Filev, Amir H. Davarpanah, Eugene A. Berkowitz, Stefan Tigges, Scott J. Lee, Brianna L. Vey, Ali Adibi, Carlo N. De Cecco

**Affiliations:** Department of Radiology and Imaging Sciences, Emory University Hospital | Emory Healthcare, Inc., Atlanta, GA, USA; School of Electrical and Computer Engineering, Georgia Institute of Technology, Atlanta, GA, USA; School of Electrical and Computer Engineering, Georgia Institute of Technology, Atlanta, GA, USA; School of Electrical and Computer Engineering, Georgia Institute of Technology, Atlanta, GA, USA; Department of Radiology and Imaging Sciences, Emory University Hospital | Emory Healthcare, Inc., Atlanta, GA, USA; Department of Radiology and Imaging Sciences, Emory University Hospital | Emory Healthcare, Inc., Atlanta, GA, USA; Department of Radiology and Imaging Sciences, Emory University Hospital | Emory Healthcare, Inc., Atlanta, GA, USA; Department of Radiology and Imaging Sciences, Emory University Hospital | Emory Healthcare, Inc., Atlanta, GA, USA; Department of Radiology and Imaging Sciences, Emory University Hospital | Emory Healthcare, Inc., Atlanta, GA, USA; Department of Radiology and Imaging Sciences, Emory University Hospital | Emory Healthcare, Inc., Atlanta, GA, USA; Department of Radiology and Imaging Sciences, Emory University Hospital | Emory Healthcare, Inc., Atlanta, GA, USA; Department of Radiology and Imaging Sciences, Emory University Hospital | Emory Healthcare, Inc., Atlanta, GA, USA; School of Electrical and Computer Engineering, Georgia Institute of Technology, Atlanta, GA, USA; Department of Radiology and Imaging Sciences, Emory University Hospital | Emory Healthcare, Inc., Atlanta, GA, USA

## Abstract

**Objective:**

The purpose was to evaluate reader variability between experienced and in-training radiologists of COVID-19 pneumonia severity on chest radiograph (CXR), and to create a multireader database suitable for AI development.

**Methods:**

In this study, CXRs from polymerase chain reaction positive COVID-19 patients were reviewed. Six experienced cardiothoracic radiologists and two residents classified each CXR according to severity. One radiologist performed the classification twice to assess intraobserver variability. Severity classification was assessed using a 4-class system: normal (0), mild (1), moderate (2), and severe (3). A median severity score (Rad Med) for each CXR was determined for the six radiologists for development of a multireader database (XCOMS). Kendal Tau correlation and percentage of disagreement were calculated to assess variability.

**Results:**

A total of 397 patients (1208 CXRs) were included (mean age, 60 years SD ± 1), 189 men). Interobserver variability between the radiologists ranges between 0.67 and 0.78. Compared to the Rad Med score, the radiologists show good correlation between 0.79–0.88. Residents show slightly lower interobserver agreement of 0.66 with each other and between 0.69 and 0.71 with experienced radiologists. Intraobserver agreement was high with a correlation coefficient of 0.77. In 220 (18%), 707 (59%), 259 (21%) and 22 (2%) CXRs there was a 0, 1, 2 or 3 class-difference. In 594 (50%) CXRs the median scores of the residents and the radiologists were similar, in 578 (48%) and 36 (3%) CXRs there was a 1 and 2 class-difference.

**Conclusion:**

Experienced and in-training radiologists demonstrate good inter- and intraobserver agreement in COVID-19 pneumonia severity classification. A higher percentage of disagreement was observed in moderate cases, which may affect training of AI algorithms.

**Advances in knowledge:**

Most AI algorithms are trained on data labeled by a single expert. This study shows that for COVID-19 X-ray severity classification there is significant variability and disagreement between radiologist and between residents.

## Introduction

COVID-19 disease is currently recognized as a global pandemic with more than 100 million cases worldwide. Medical imaging techniques such as chest radiographs (CXRs) and CT play an important role in COVID-19 diagnosis.^1–4^ CXRs are often the first imaging resource available.^5,6^ The American College of Radiology (ACR) suggests using portable CXRs in ambulatory care facilities in its recent COVID-19 guidelines for the use of chest imaging.^7^ The British Medical Journal (BMJ) recommends the use of CXR in all patients with suspected COVID 19 pneumonia.^8^ In addition, CXRs are increasingly used to evaluate disease severity and for prognostic purposes.^9–12^ These studies show that CXR is useful for classifying disease severity and predicting patient outcome.

To our knowledge, very few studies has assessed inter- and intraobserver variability among experienced and resident radiologists for detection of typical and atypical pneumonia.^13^ A lack of information on the variability of COVID-19 severity classification may affect current clinical settings and treatment plans and may limit the development of effective AI algorithms to assist radiologists with this task. A study by Li et al showed in a multiradiologist reading study that AI can improve interrater agreement significantly.^14^ AI can play a crucial role in automatic assessment and monitoring of COVID-19 related pneumonia using CXRs.^9,15^ Many AI algorithms have been developed for the detection and severity classification of COVID-19 pneumonia; however most of the databases used for the training and validation lack multireader assessment and validation. For example, several AI algorithms use the same online available data sets, leading to the same systematic errors and labeling variability.^9,16^ The variability in the reference standard used leads to bias in the training phase and decreased accuracy in the validation stage when reader variability is introduced.^17^ There are a few initiatives to create multireader databases such as done for the PXS score and with the RSNA RICORD initiative.^14,18^ AI development is highly dependent on the data used for training in terms of quality, quantity and representability of the population of interest.^17,19,20^ External validation is rarely performed in (COVID-19) AI studies, but is highly desired before clinical implementation to assess accuracy compared to radiologists for the population of interest.^21^ Thus, the development of an accurate multireader labeled CXR-database will be of great utility to increase the generalization of the reference standard and to assess the real-life accuracy of an AI algorithm in comparison to multiple readers.

The purpose of this study was to evaluate the inter- and intraobserver variability between multiple experienced and in-training radiologists for severity classification of COVID-19 pneumonia on CXRs, and to create a CXR COVID-19 multireader composite score database suitable for AI algorithm development.

## Methods

### Data collection

CXRs from COVID-19 patients were retrospectively collected from January 1, 2020 to May 1, 2020 as a consecutive sample. Patients were included if they had a positive polymerase chain reaction (PCR) test for which the CXR were taken and if the posteroanterior or anterpposterior projection were of diagnostic quality. Patients were excluded for the following reasons: 1) age <18. A total of 1208 CXRs from 397 COVID-19 positive patients were included. Only posteroanterior and anteroposterior projection CXRs with optimized window settings for pulmonary evaluation were included in this study, lateral CXRs were excluded. In addition to CXRs, the following clinical data were collected for each patient; (1) age, (2) gender, (3) ethnicity, (4) days of admission and (5) mortality related to COVID-19.

The need for informed consent was waived by the institutional review board (IRB). All images were de-identified according to the Health Insurance Portability and Accountability Act (HIPAA) and hospital-specific guidelines. In addition, the date and time stamp burned into the CXRs was removed. All these images are also used for a previously published article.^15^ This prior article dealt with development of an artificial intelligence (AI) algorithm using a single reading, whereas in this manuscript, we report on the reading variability using multiple readers.

### Data labeling

Six expert board-certified cardiothoracic radiologists each with 10+ years of experience in reading CXRs were asked to label each CXR according to severity. In addition, two second-year radiology residents were asked to classify severity. One expert cardiothoracic radiologist (CNDC) performed the classification twice, with a 4 month time-interval to avoid recall bias to assess intraobserver variability.

There are few studies describing severity scores for COVID-19, however, none are validated in large generalizable cohorts.^10,11^ We chose a severity rating based on the most prevalent abnormality seen in COVID-19 pneumonia with more general descriptions of extent based on current literature and clinical findings.^1,4,12,22^

A retrospective study by Wong et al on 64 patients with PCR-confirmed COVID-19 diagnosis showed that the most common signs on CXR were consolidation (47%) and ground-glass opacities (33%).^1^ Our severity classification was focused mostly on consolidations and the extent of lung involvement, because these findings are best at predicting the effect on pulmonary function. Severity classification was performed using a 4-class system: 1, normal; 2, mild; 3, moderate; and 4, severe. Normal was defined as no lung abnormalities. Mild cases were defined as showing as opacifications in the mid and lower lung zones, patchy and often peripheral in nature involving less than 25% of the most severely affected lung. Moderate cases were defined as bilateral as opacifications involving the peripheral mid and lower lung zones with approximately >=25% and <=50% involvement of the most severely affected lung. Severe cases were defined as patchy bilateral opacification (air space opacities or consolidations) with involvement of more than 50% of the most severely affected lung. Before the start of the reading study, all readers received information about the classification and had access to multiple examples of each class.

**Figure 1.**
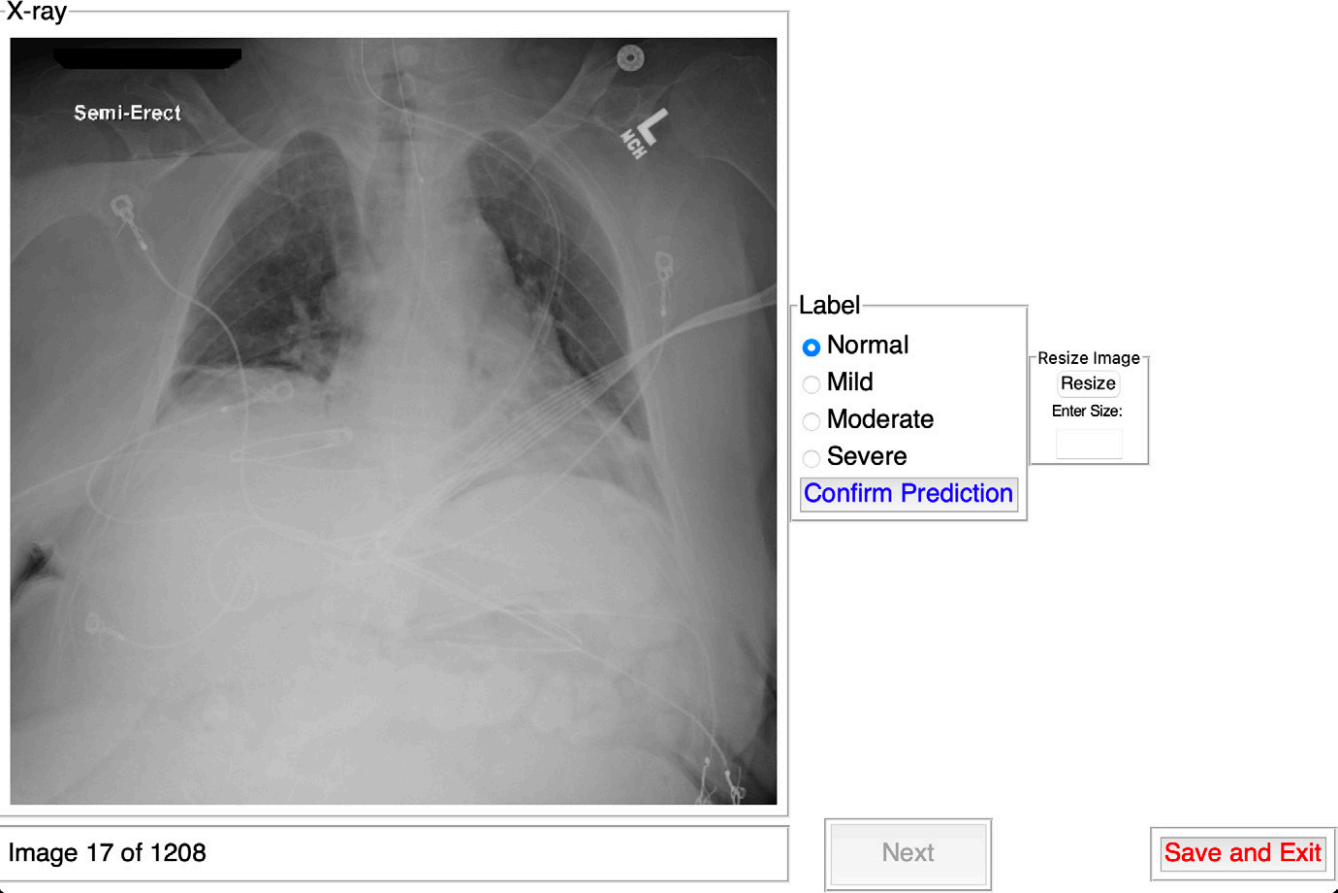
Labeling program that shows readers CXRs in random order and allows them to label each CXR according to disease severity. The program allows for resizing of the window and to save the labeling whenever the reader desires. CXR, chest radiograph.

Data labeling was performed using an in-house platform that randomized the CXRs for each reader. The platform (Figure 1) allowed optimization of the window size to the evaluator’s screen size. Readers classified each CXR and were asked to confirm their classification before moving on to the next case. Readers were blinded to clinical data and outcomes. Readings were performed on each specialist’s preferred computer at the convenience of the reader. The labeling did not have to be performed in one sitting.

### Statistical analysis

For quantitative analysis of the severity classification, the classes were transformed into a scale-wise parameter ranging from 0 to 3 (0 = normal, 1 = mild, 2 = moderate and 3 = severe). A median score of all radiologists’ scores was calculated (Rad Med) as a composite gold-standard. The percentage of agreement and Kendall’s Tau coefficient were used to calculate the inter- and intraobserver variability. Kendall’s Tau coefficients were calculated between all radiologists and residents and for the average classification of all six radiologists (Rad Med) compared to each individual reader. We investigated the class-differences between the radiologists and residents for each CXR and evaluated in which cases multiple readers and multiple readings differed. Coefficient classes were defined as: poor agreement/high variability (τ: <0.20), fair agreement/moderate variability (τ: 0.21 to 0.40), moderate agreement/fair variability (τ: 0.41 to 0.60), good agreement/low variability (τ: 0.61 to 0.80) and very good agreement/very low variability (τ: >0.81). High reading variability is associated with low agreement and results in lower correlation coefficients. In addition, non-parametric χ^2^ or Kruskal–Wallis tests were used to assess differences in clinical variables between the different groups. To investigate the differences in the labels of the radiologists, we select five readers and consider their medians as the ground truth label and calculate the confusion matrices for others. We repeat this process by permuting the radiologists in the median and take the average and standard deviation of the values on the confusion matrix. A statistical package (SPSS, v. 26) was used for all data analysis. A *p*-value < 0.05 was considered statistically significant.

## Results

Because of image quality 19 CXRs were deleted, 1 CXR was deleted due to windowing issues. The other 18 images were excluded because essential parts of the lung fields were not visible in the image (*e.g.* patient positioning or obstruction by external equipment) The final data set includes 1208 images from 397 patients with a mean age of 60 (SD = 16 years), of which 50% were male. The majority of patients were African American (297 (75%)). Table 1 shows the demographics for the 397 patients. According to the Rad Med scoring (average of six radiologists’ classification scores), this data set of 1208 CXR consists of 142 (11%) normal, 359 (30%) mild, 305 (25%) moderate and 402 (33%) severe cases.

**Table 1.** Patient demographics

	Patients *n =* ***397***	CXRs *n =* ***1208***
Age (years)	60 (16)	62 (15)
Gender (male)	198 (50%)	662 (55%)
Ethnicity		
*Caucasian*	52 (13.1%)	142 (12%)
*African American*	297 (75%)	952 (79%)
*Asian*	6 (2%)	9 (1%)
*Hispanic*	5 (1%)	6 (1%)
*Unknown*	36 (9%)	99 (9%)
BMI (kg/m^2^)	35 (30)	36 (30)
Days of admission (days)	10 (12)	17 (14)
Deceased	58 (15%)	298 (25%)

BMI, body mass index; SD, standard deviation.

Data are given as mean (SD) or as n (%).

### Interobserver variability

**Figure 2.**
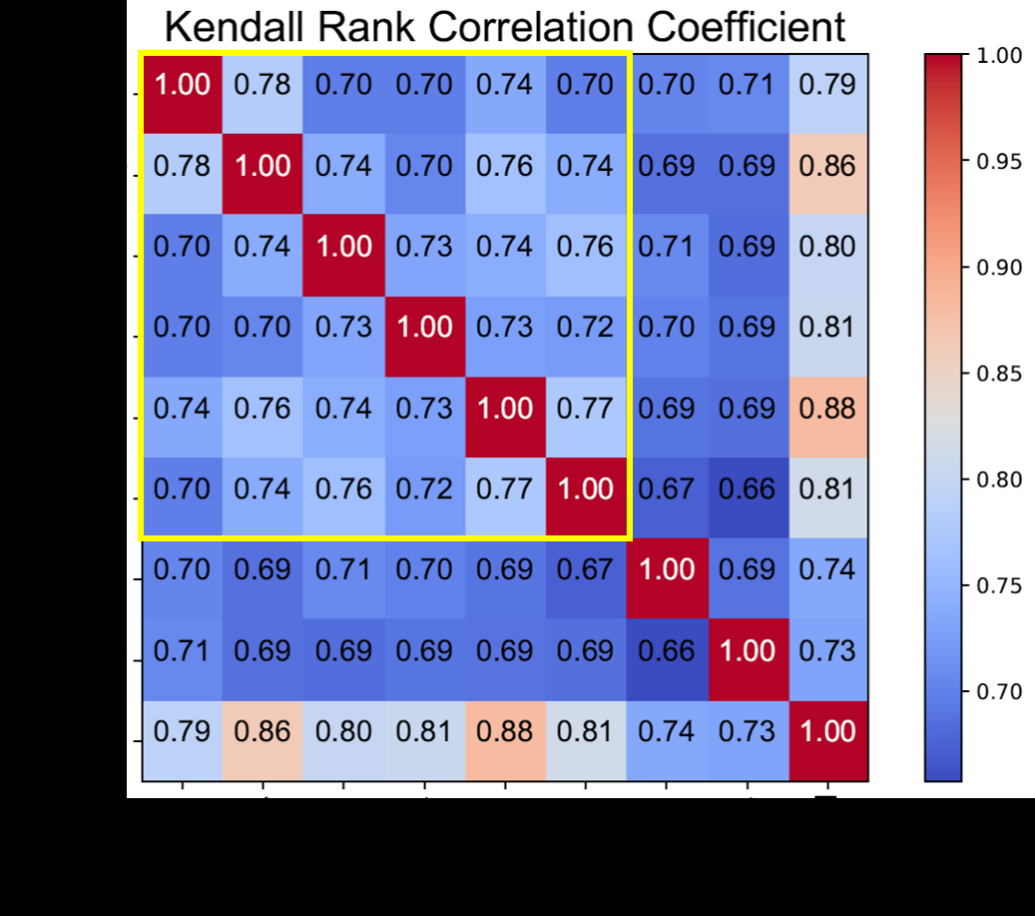
Visualization of the Kendall rank correlation coefficients of the six radiologists (Rad 1–Rad 6; yellow square) and two residents (Res 1 and Res 2). The Rad Med row represents the correlation of each reader with the median classification of all six radiologists (Rad Med)

Figure 2 shows all interobserver variabilities between the six radiologists and the two residents. In addition, the interobserver variability between the median score of the radiologists (Rad Med) and the individual radiologists and residents are shown.

The interobserver variability between the six radiologists is low and ranges between 0.67 and 0.78, indicating good agreement. Relative to the median score of all radiologist, the six radiologists show correlation coefficients ranging between 0.79 and 0.88, reflecting low variability. The two residents show an interobserver variability of 0.66 between each other and a range between 0.69 and 0.71 with the six radiologists. In relationship to the Rad Med score, the two residents show a correlation range between 0.73 and 0.74. In all cases, the residents show slightly lower correlation coefficients compared to experienced radiologists.

**Figure 3.**
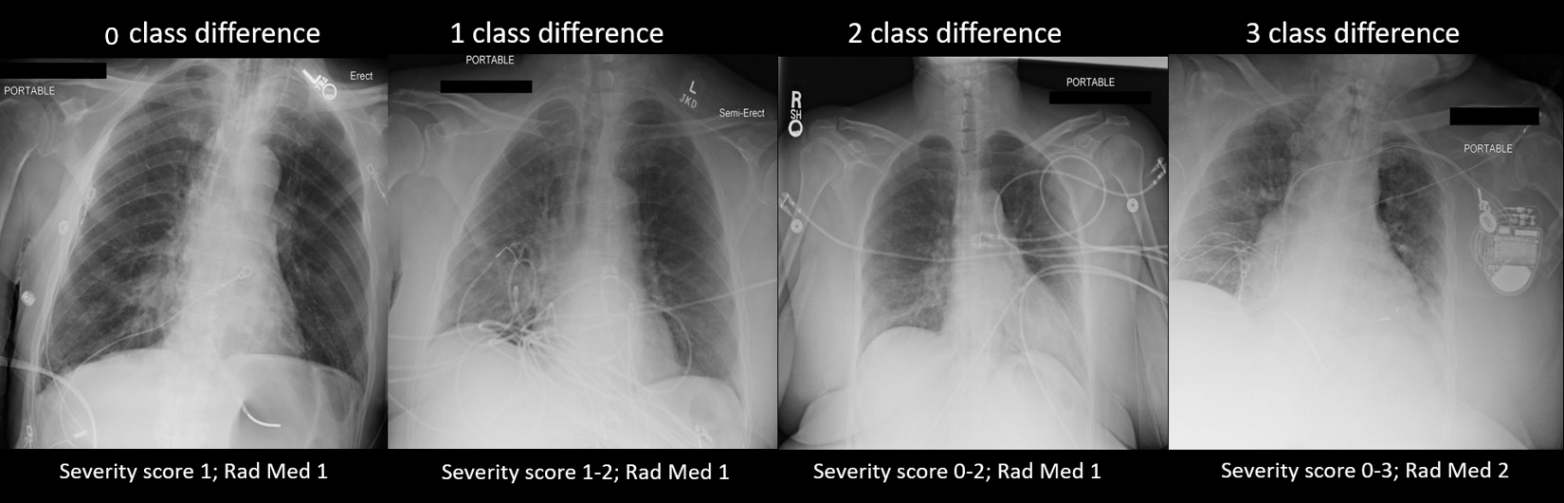
Interobserver variability—examples of 0, 1, 2, and 3 class-difference in severity labeling by six radiologists. The severity score range is given below the images as well as the median value of all radiologists‘ combined scoring (Rad Med)

Of the 1208 CXRs evaluated by the six radiologists, 220 (18%) were classified concordantly. For 707 (59%), 259 (21%) and 22 (2%) CXRs, there was a maximum class-difference of 1, 2, or 3 classes, respectively. Figure 3 shows examples of discordant cases with 0, 1, 2 or 3 class-difference. The only demographic that was different between the groups of class-differences was age (*p* < 0.001) (Table 2). However, there is a difference in median severity scores (Rad Med) between the class-difference groups (*p* = 0.042). There is a lower frequency of 0 and 3 severity scores in the 2 and 3 class-difference group compared to the (0 and 1 class-difference groups. There is an increase in 1 and 2 severity scores in the 2 and 3 class-difference groups compared to the 0 and 1 class-difference groups. Comparison of Rad Med scores and the distribution of class-differences between each class shows, a significant difference in class-difference between each score (*p* < 0.001) (Table 2). The moderate (2) class only show agreement in 6%, compared to the severe (3) class with 48%), whereas the mild (1) cases show a 3 class-difference in 60% of cases.

**Table 2.** Overview of class-differences in severity labeling by radiologists

	0 class-difference *n = 220* (*18%*)	1 class-difference *n = 707* (*59%*)	2 class-difference *n = 259* (*21%*)	3 class-difference *n = 22* (*2%*)	*p*-value
Severity score					
Median	2 (1–3)	2 (1–3)	2 (1–2)	1 (1–2)	0.042
Frequencies					<0.001
*Normal (0)*	44 (20%)	90 (13%)	8 (3%)	0 (0%)	
*Mild (1)*	57 (26%)	214 (30%)	75 (29%)	13 (60%)	
*Moderate (2)*	13 (6%)	153 (22%)	132 (51%)	7 (32%)	
*Severe (3)*	106 (48%)	707 (35%)	44 (17%)	2 (9%)	
Age (years)	59 (17)	62 (16)	65 (12)	67 (11)	<0.001
Gender (male)	121 (55%)	406 (57%)	125 (48%)	10 (46%)	0.282
Ethnicity					0.329
*Caucasian*	32 (15%)	85 (12%)	22 (9%)	3 (14%)	
*African American*	158 (72%)	565 (80%)	212 (82%)	17 (77%)	
*Asian*	2 (1%)	5 (1%)	2 (1%)	0 (0%)	
*Hispanic*	2 (1%)	4 (1%)	0 (0%)	0 (0%)	
*Unknown*	26 (12%)	48 (7%)	23 (9%)	2 (10%)	
BMI (kg/m^2^)	34.0 (20)	37 (35)	36 (25)	36 (10)	0.738
Days of admission (days)	17 (14)	17 (15)	16 (11)	16 (13)	0.633
Deceased	62 (30%)	172 (24%)	59 (22.8%)	5 (23%)	0.900

CXR, chest radiograph; SD, standard deviation.

Data are given as mean (SD) or as n (%) for each CXR included in the data set. *p*-value represents the comparison of the 4 class-difference groups with a *p* < 0.05 considered as significant.

Of the 1208 CXRs evaluated by the 2 residents, 750 (62%) were classified concordantly, 411 (34%) and 47 (4%) had a 1 and 2 class-difference, respectively. There were no cases where the severity was scored with a 3-class-difference between the two residents. In only 66% (Res 1) and 65% (Res 2) of cases were Rad Med and residents’ score similar; in 31% (Res 1) and 32% (Res 2) of cases the score had a one class-difference. In 594 (50%) CXRs, the median score of the residents and the radiologists was the same; in 578 (48%) and 36 (3%) CXRs there was a 1 and 2 class median difference between residents and radiologists median scores, respectively.

**Figure 4.**
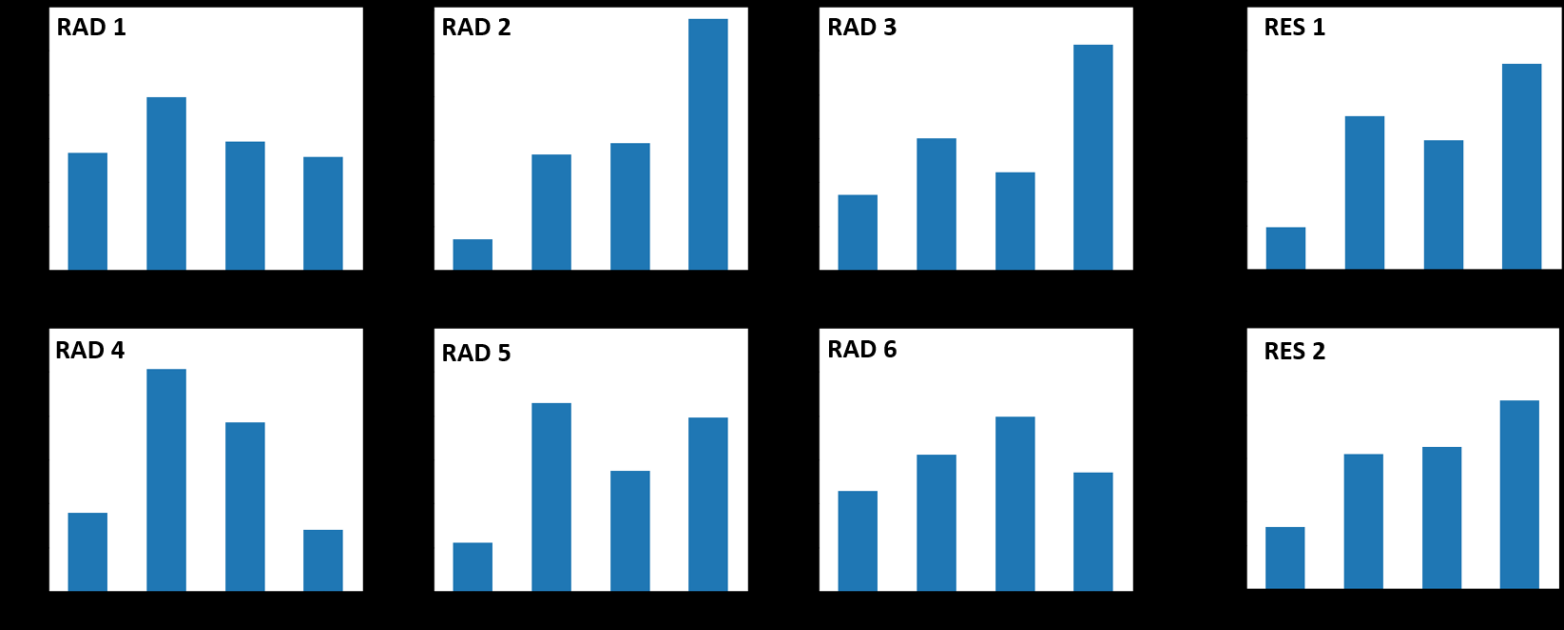
Distribution of the severity classes for the readers. As it is shown, there is a significant difference between the distribution of the labels among readers. For instance, Rad 2 and Rad 3 are skewed towards severe class while Rad 1, Rad 6 have a close to uniform distribution of labels.

Not only do the severity classification differ per case, Figure 4 shows the severity classification distribution for each radiologist showing a variability in distribution between radiologist and between residents and radiologist.

**Figure 5.**
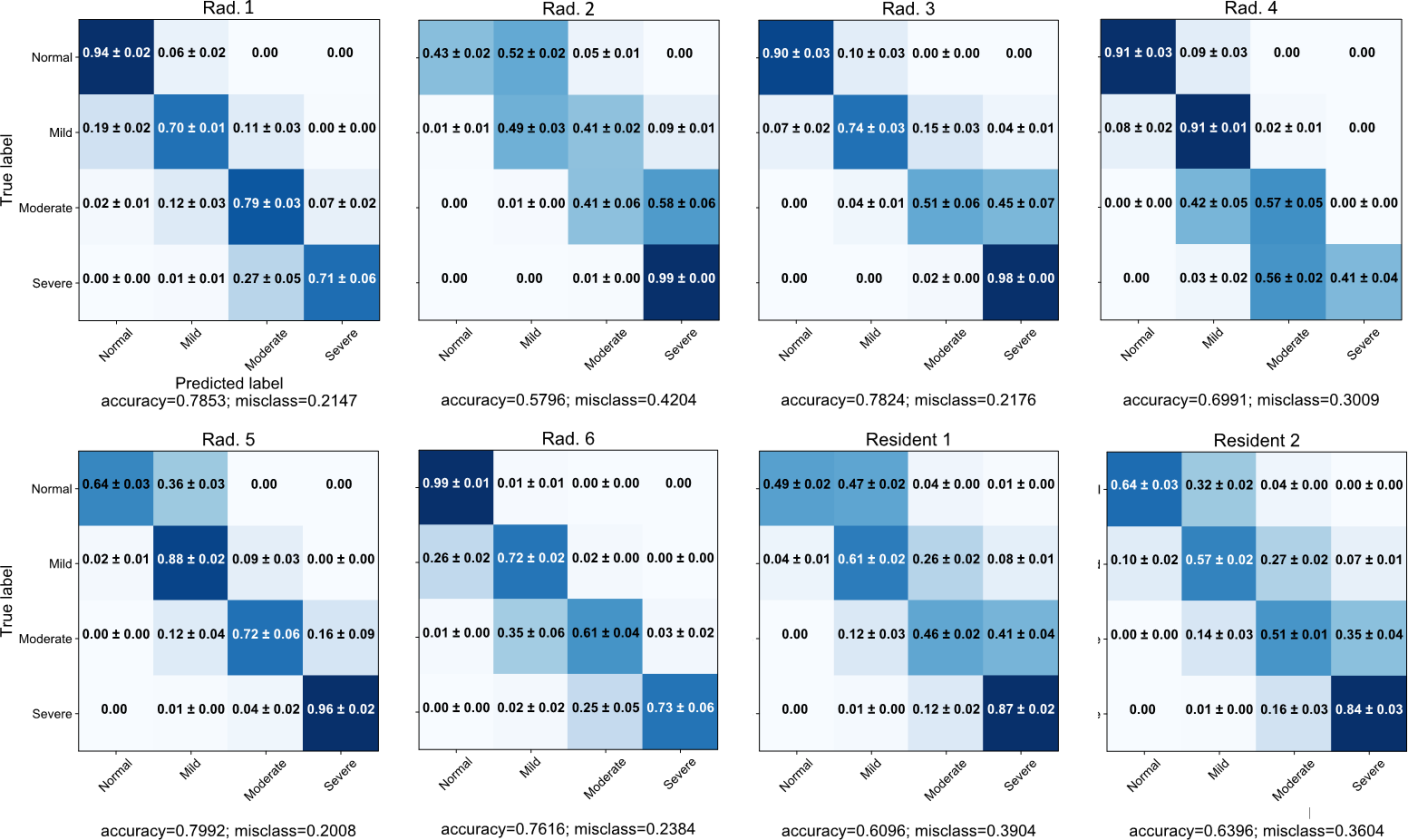
Confusion matrices of radiologist and residents. For each radiologist, the other five radiologist serve as the reference standard. These matrices show that performance metrics are affected by changes in the reference standard caused by the use varying sets of radiologists.

As it is shown in Figure 5, the average sensitivity of the radiologists can be as low as 0.43, 0.49, 0.41, and 0.41 for the normal, mild, moderate, and severe classes, respectively. Standard deviations differ per class and radiologist between 0.0 and 0.09. The confusion matrices show that there is depending on the radiologist an over or underestimation takes place. Radiologists 2 and 5 show signs of overestimation while radiologists 4 and 6 show signs of underestimation of COVID-19 severity. Highest variation is shown around the moderate severity class for both over- and underestimation. Both residents show that they overestimate COVID-19 severity for each class.

### Intraobserver variability

**Figure 6.**
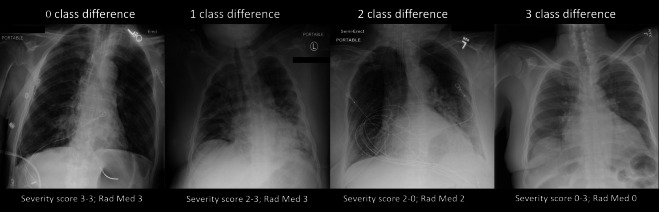
Intraobserver variability. Examples of 0, 1, 2 and 3 class-differences in severity labeling. The severity scores of both readings are given below the images as well as the median value of all six radiologists combined scoring (Rad Med).

One radiologist evaluated all 1208 images twice, resulting in a Kendall’s Tau coefficient of 0.77 representing intraobserver variability. In 833 (69%) of CXRs, there was a 0 class-difference in severity labeling between the first and second read. In 367 (30%), 7 (1%), and 1 (0%) there was a 1, 2, and 3 class-difference in severity labeling, respectively. None of the demographics was significantly different between class-difference groups. Figure 6 shows an example of discordant cases with 0, 1, 2, or 3 class-difference. Table 3 shows an overview of the class-differences between the inter- and intraobserver scores. A majority of the CXRs shown similar (433 (35%)) severity classifications or a 1 class-difference (583 (48%)) between the inter- and intraobserver analysis.

**Table 3.** Overview of class discordance between inter- and intraobserver severity class scores

		Class-differences intraobserver				Total
		0	1	2	3	
**Class-differences interobserver**	**0**	195	25	0	0	220
	**1**	462	243	1	1	707
	**2**	160	94	5	0	259
	**3**	16	5	1	0	22
Total		833	367	7	1	1208

## Discussion

This study investigates the CXR inter- and intraobserver variability among experienced and radiologists in training of COVID-19 pneumonia severity classification. In addition, a multireader composite score for COVID-19 severity (Rad Med) has been created for the development of AI algorithms (XCOMS database). Our results demonstrate that experienced radiologists show good interobserver variability; while the two residents showed slightly lower correlation coefficients. Although good variability was shown, comparing the experienced radiologist’s scores, there was classification disagreement in 82% of all CXRs, with 59% showing a 1 class-difference. Discordance of severity classification was not related to any demographic features, besides age, but was significantly influenced by severity level. As expected, normal and severe classes resulted in the highest agreement between readers (68% agreement), whereas moderate cases had the lowest agreement (6%) and the mild cases showed a 3 class-difference in 60% of CXRs.

Considering that CXR utilization is recommended by many scientific societies for the diagnosis and management of COVID-19 pneumonia, knowing the inter- and intraobserver variability is relevant both to daily clinical practice and AI development.^7,8^ To our knowledge, few published studies exist investigating the inter- and intraobserver variability among radiologists for the detection of community acquired pneumonia using CXR. Moncada et al found that for community acquired pneumonia, variability is 0.72 for interobserver variability and ranges between 0.61 and 0.85 for intraobserver variability using a semi-quantitative approach.^13^ In our study on COVID-19 pneumonia, we observed a comparable inter- and intraobserver variability using a similar semi-quantitative analysis. The advantage of using a clearly defined class-based reading is to decrease variability compared to a descriptive analysis as used in clinical practice.

One earlier study on the effect of AI on radiologist interrater agreement for COVID-19 severity classification on 154 CXRs, using multiple radiologist with thoracic or emergency subspecialties, showed low interrater agreement (Fleiss *k* = 0.40), where thoracic radiologist show a slightly higher agreement of 0.50 compared to emergency radiologist with 0.25.^14^ This also indicates, similarly as in our study, that experience decreases variability.

A study by Cohen et al on the prediction of COVID-19 pneumonia severity on CXR using deep learning also performed a multireader analysis, using two radiologist and one resident on 94 CXRs. They showed interobserver agreements of 0.45 using an opacity score and 0.71 for an extent score. However, they used a limited sample-size, and grouped experienced and in-training radiologists, limiting validity of their results on reader performance.^12^

In our study, the interobserver variability is good (0.69–0.81), and as expected was higher for experienced radiologists compared to residents. However, in only 18% of cases did all six experienced radiologists agree on the severity classification. In 59%, there was only a 1 class-difference in severity labeling and in 23%, there was a 2 or more class-difference in severity labeling between the six radiologists. These differences are unlikely to affect diagnosis and clinical management, although it may have a major effect on the training and results of an AI algorithm. In the 142 normal cases (according to Rad Med score), only 31% of cases showed a 0 class-difference between radiologists. This indicates that for 69% of normal X-rays radiologist show a difference of opinion on COVID-19 severity. The differentiation between normal and abnormal could have the greatest impact on decision-making and management. However, our results also show that in 63% the normal cases show only 1 class-difference and were mostly classified as normal or mild. The cases demonstrating the highest differences in severity classification, were mostly from the intermediate groups (mild and moderate classes) as determined by the median classification score of all six radiologists.

Evaluating the accuracy of each radiologist compared to the other radiologist, the high standard deviations (*e.g.* 0.09 for moderate/severe class for Rad 5) shows that depending on the selection of different readers, the reference standard (*i.e.* median of 5 readers) can significantly change and affect the performance metrics. Therefore, this issue should be considered during the training and evaluation of an AI approach for severity assessment and diagnosis of the disease. Studies, however, have showed that AI can also help decrease the variability between radiologist, as shown by Li et al, where AI increased the Fleiss k from 0.40 to 0.66 by using an AI model for COVID-19 severity.

Intraobserver variability was 0.77 with 833 (69%) of CXRs showing concordant readings between the first and second read. In only eight of cases (1%), was there a greater than 1 class-difference between readings, indicating excellent agreement. This clearly demonstrates the need for using a multireader reference standard for AI development as two readings by a single experienced radiologist shows disagreement in 31% of cases.

In-training radiologists demonstrate a slightly higher variability in severity classification compared to experienced radiologists, indicating that experience decreases severity classification variability. In only 66% (Res 1) and 65% (Res 2) of cases were Rad Med and residents’ score similar; in 31% (Res 1) and 32% (Res 2) of cases, the score had a 1 class-difference. The confusion matrices show that in-training radiologist overestimate severity for each class compared to the radiologist. This database can be used for educational purposes to increase training for COVID-19-related pneumonia assessment.

Most available AI studies for COVID-19 detection have been developed using single-reading training sets, raising concerns about correct data labeling and variability in the reference standard. For example, the ChestCXR14 data set (containing 30,805 CXRs) from the National Institutes of Health Clinical is a commonly used database for AI training. However, this data set may not be fit for training medical AI algorithms to do diagnostic work, mostly due to issues with correct labeling.^23^ A study by Pooch et al raises similar concerns: they showed that 90% of lung cancer misdiagnosis occurs on CXRs, often due to reader error.^24^ They trained and tested multiple neural networks on several CXR databases, resulting in accuracies 10–30% lower than values originally reported. Incorrect label extraction was the most common error. Creating an accurate imaging data set using multiple experienced radiologists is a time-consuming and costly endeavor; a composite labeling approach as in our case can result in a more robust set for training and provide further insight in the labeling process to create curated and transparent data sets for AI training and external validation. There are a few initiatives out there to create multireader databases such as the RSNA International COVID-19 Open Radiology Database,^18^ which consists of 1000 CXRs contributed from four international sites with labels from three radiologists. XCOMS is unique by using a large amount of CXRs with labels from six radiologist and two residents, enabling AI training and validation to see whether AI can outperform radiologist and residents while considering reference standard variability. The XCOMS database is currently being expanded to reach 5000+ cases, including severity scores per lobe. After completion of the data set, it will be made available for AI algorithm development and for educational purposes.

### Limitations

There are several limitations of this trial. First, the study includes CXRs from patients with a positive PCR COVID-19 test, independent from their medical history. The data set could therefore contain images of patients with pre-existing pulmonary conditions, such as lung cancer or emphysema. However, this approach was chosen to represent a clinical sample of COVID-19 patients. In future studies, the medical history of each patient will be taken into account. Second, a qualitative 4-class severity score was developed and utilized for classification, other utilized severity scores may produce different results. Future studies should investigate the reproducibility and usefulness of other severity scores, including fully quantitative methods. Finally, only one reader was used to assess intraobserver variability, larger studies are needed to confirm these results and allow further generalizability.

## Conclusion

This study shows that, although experienced and in-training radiologists demonstrate good correlation in COVID-19 pneumonia CXR severity classification, there was significant disagreement in severity classification. A higher percentage of disagreement was observed in moderate cases, which is unlikely to affect clinical decisions, but may affect the training of AI algorithms. The developed multireader composite severity score database may overcome this limitation.
